# Thermosensitive In Situ Gelling Poloxamers/Hyaluronic Acid Gels for Hydrocortisone Ocular Delivery

**DOI:** 10.3390/gels10030193

**Published:** 2024-03-12

**Authors:** Fabrizio Villapiano, Teresa Silvestri, Camilla Lo Gatto, Danilo Aleo, Virginia Campani, Sossio Fabio Graziano, Concetta Giancola, Federica D’Aria, Giuseppe De Rosa, Marco Biondi, Laura Mayol

**Affiliations:** 1 Department of Pharmacy, University of Naples Federico II, D. Montesano St. 49, 80131 Naples, Italy; fabrizio.villapiano@unina.it (F.V.); teresa.silvestri@unina.it (T.S.); camilog994@gmail.com (C.L.G.); virginia.campani@unina.it (V.C.);; 2 Medivis Srl, Carnazza St. 34/C, 95030 Tremestieri Etneo, Catania, Italy; danilo.aleo@medivis.it; 3 Interdisciplinary Research Centre on Biomaterials (CRIB), Piazzale Tecchio 80, 80125 Naples, Italy; laumayol@unina.it; 4 Department of Advanced Biomedical Sciences, University of Naples Federico II, S. Pansini St. 5, 80131 Naples, Italy

**Keywords:** ophthalmic drug delivery, hyaluronic acid, hydrogels, DSC, thermosensitive systems, in situ forming drug reservoir, hydrocortisone release kinetics

## Abstract

This study endeavored to overcome the physiological barriers hindering optimal bioavailability in ophthalmic therapeutics by devising drug delivery platforms that allow therapeutically effective drug concentrations in ocular tissues for prolonged times. Thermosensitive drug delivery platforms were formulated by blending poloxamers (F68 and F127) with low-molecular-weight hyaluronic acid (HA) in various concentrations and loaded with hydrocortisone (HC). Among the formulations examined, only three were deemed suitable based on their desirable gelling properties at a temperature close to the eye’s surface conditions while also ensuring minimal gelation time for swift ocular application. Rheological analyses unveiled the ability of the formulations to develop gels at suitable temperatures, elucidating the gel-like characteristics around the physiological temperature essential for sustained drug release. The differential scanning calorimetry findings elucidated intricate hydrogel–water interactions, indicating that HA affects the water–polymer interactions within the gel by increasing the platform hydrophilicity. Also, in vitro drug release studies demonstrated significant hydrocortisone release within 8 h, governed by an anomalous transport mechanism, prompting further investigation for optimized release kinetics. The produced platforms offer promising prospects for efficacious ocular drug delivery, addressing pivotal challenges in ocular therapeutics and heralding future advancements in the domain.

## 1. Introduction

The eye presents unique challenges in terms of its anatomical and physiological nature, as well as its defense mechanisms, thus rendering drug delivery to ocular tissues a cumbersome task due to issues associated with bioavailability. Topical instillation of drugs via eye drops is the primary route of administration for the treatment of various ocular disorders affecting the anterior segment of the eye, including dry eye disease, conjunctivitis, uveitis, diabetic macular oedema and postoperative inflammation [1]. However, conventional pharmaceutical formulations, such as solutions and suspensions, have many drawbacks. These include rapid precorneal elimination, gravity drainage, normal tear turnover, enzymatic metabolism, nasolacrimal drainage, conjunctival absorption, and lack of controlled release and bioadhesive properties [2]. The residence time of most conventional eye solutions is 5–25 min, with only 1–10% of the topically applied drug being absorbed; moreover, an important part of the drug is absorbed systemically, thereby causing adverse effects [3]. The limited permeability of the ocular membranes also contributes to the low absorption of the drugs, determining the short duration of the therapeutic effect and making a frequent dosing regimen necessary. On the other hand, the instillation of highly concentrated eye drops can cause adverse effects and cellular damage to eye tissues [4]. Alternatively, when facing diseases of the posterior eye segment, such as uveitis, diabetic retinopathy, macular edema, and age-related macular degeneration, an effective concentration of the active molecule(s) at the target site for long times is required. In such cases, intravitreal injection offers distinct advantages since the drug is directly inserted into the vitreous [1,5]. However, drug distribution in the vitreous is nonuniform: small molecules can rapidly distribute through the vitreous, whereas the diffusion of larger molecules is restricted. Actually, the transport of drugs within the vitreous body depends on its viscoelastic properties, which, in turn, depend on the age, gender and pathology of the patient [6]. Moreover, intravitreal injection is associated to non-desired adverse effects, such as cataracts, retinal detachment, and hemorrhages. The occurrence of undesired effects of course increases with an increasing number of injections [7]. In this context, the use of an in situ forming hydrogel acting as an analogue of a healthy vitreous body and able to sustain the release of the drug(s) may be particularly useful, being able to reduce the frequency of administrations and, therefore, the occurrence of undesired effects. This approach may also be advantageous for topical instillation, with the goal of enhancing the bioavailability of the drug(s). The hydrogel is easily administered in its liquid state via standard eyedrops, and its resistance to drainage is expected to increase once it has gelled.

This panorama highlights the potential of in situ gelling ophthalmic drug delivery systems based on heat-sensitive amphiphilic block copolymers such as poloxamers, which have been extensively investigated for their phase-reverse thermal gelation. Poloxamers are triblock copolymers composed of poly(ethylene oxide)–poly(propylene oxide)–poly(ethylene oxide) (PEO–PPO–PEO), which undergo self-assembly through micellization. This intricate process is dictated by two crucial parameters, namely the critical micelle concentration and the critical micelle temperature, as schematically shown in the graphical abstract [8,9]. The PEO/PPO molar ratio and the molecular weight of the PEO and PPO blocks primarily determine these features. Moreover, due to their amphiphilic properties, poloxamers can interact with both hydrophilic and hydrophobic drugs, which has led to their application as controlled release systems. These gel formulations are relatively simple to manufacture and widely utilized in the pharmaceutical field as they are generally regarded as safe (GRAS) excipients.

Nevertheless, poloxamer-based gels for drug delivery have major limitations, such as low stability, poor mechanical properties, and short residence times due to rapid dissolution in the biological milieu [10]. In previous studies, we have proposed a novel approach that involves blending poloxamers with a mucoadhesive polymer, such as hyaluronic acid (HA), to enhance the mechanical properties and the mucoadhesive strength of poloxamer-based gels [10,11]. HA is a naturally occurring biodegradable, highly biocompatible and mucoadhesive polysaccharide, and also a main constituent of the extracellular matrix of connective tissues. These properties make HA an attractive polymer for the formulation of drug delivery applications [11,12,13].

In the present work, novel thermosensitive drug delivery platforms with optimized mechanical properties, gelling temperature and time were devised and produced. The formulations were obtained by blending two different poloxamers (F127 and F68) with HA in different concentrations. The resulting hydrogels were then loaded with hydrocortisone (HC) by simple dispersion. HC has been vastly utilized in the ophthalmic domain by virtue of its pronounced anti-inflammatory effect, both in vitro and in vivo, against dry eye disease [14], and it also brings about an improvement in the symptoms of ocular surface disease in patients with and without primary open-angle glaucoma [15]. Unfortunately, HC is sparingly soluble in water, posing significant challenges for its ocular administration since the drug easily precipitates [16]. Thus, it is of great interest to investigate the possibility of administering native HC by using solubility promoters, such as poloxamers, which are able to self-assemble into micelles. The obtained gels were characterized for their morphological, physico-chemical and thermodynamic features by means of scanning electron microscopy (SEM), Fourier transform infrared spectroscopy (FTIR) and differential scanning calorimetry (DSC). Moreover, the gels were optimized in terms of their rheological properties and gelation temperature/time. Finally, the release kinetics of HC from the gels were assessed by spectrophotometric assay.

## 2. Results and Discussion

### 2.1. Hydrogel Selection and Characterization

Table 1 summarizes the compositions of the 14 formulations tested in this work. After an extensive formulation analysis, only three formulations were deemed suitable based on their desirable gelling properties. Specifically, these formulations were chosen for their ability to form a gel within the desired temperature range of 35–37 °C, mirroring the physiological conditions of the eye’s surface. Moreover, a minimal gelation time was needed for ocular application, ensuring a suitable waiting time before achieving the desired consistency. The fluid nature of the selected formulations at room temperature presents a further advantage: this characteristic allows for straightforward sterilization through filtration while in their liquid state, a critical requirement for industrial scale-up. The selected gels are marked with an asterisk. Additionally, multi-dose vials with contamination prevention systems are available nowadays. The administration of a liquid system from these multi-dose systems is possible, provided the formulation viscosity limitations are met. The pH of the pre-gels was found to be around 7.6 in all cases, which is well within the tolerability range for ocular delivery. The average osmolarity of the freshly produced gels was found to be consistent across all the samples, as illustrated in Figure S1, which shows that the osmolarity values vary in a quadratic fashion based on the poloxamer concentration. The extrapolated osmolarity value for the undiluted gel was found to be 1267 mOsm/L. Although the observed osmolarity value is pretty high, it is important to note that HC administration carries a risk of inducing ocular hypertension [17]. Thus, in the case of topical administration of HC-loaded poloxamer-based gels, this effect may be mitigated. Indeed, the gel can facilitate the outward movement of fluids from the vitreous body, counteracting the increase in intraocular pressure induced by HC.

**Figure 1:**
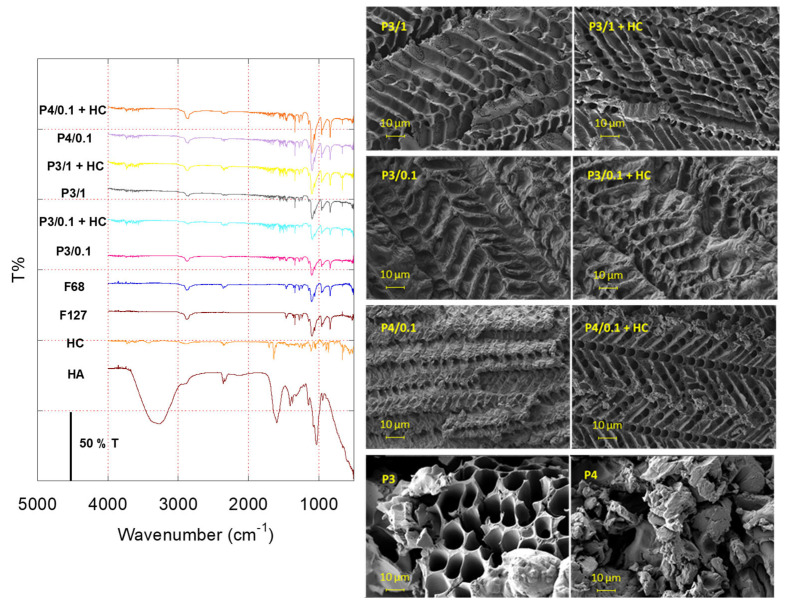
Fourier transform infrared spectra (FTIR) of raw materials and selected hydrogels (**left**). Scanning electron microscopy (SEM) micrographs of freeze-dried hydrogels with (**right**) or without HC (**left**).

Figure 1 reports the FTIR spectra of both the raw materials and hydrogels. Analysis of the FTIR traces reveals that the characteristic peaks corresponding to the functional groups of the raw materials remain unchanged after hydrogel formation. This suggests a minimal interaction between the drug and hydrogel components. Notably, the low-intensity peaks observed in the hydrogel spectra within the same region as the raw materials hint at the presence of HC and HA 830 within the samples. However, their signals are subdued due to their lower concentrations compared to the poloxamers in the formulation.

SEM micrographs of freeze-dried hydrogels are also shown in Figure 1. All the presented pictures show a particular and distinct network structure, a kind of herringbone fabric, a coated and ordered arrangement attributed to the stacking of poloxamer micelles and the presence of a hyaluronic acid coating. The morphological evidence can be related to variations in the poloxamer and HA content, which significantly impact the hydrogel surface. An increase in the HA content (P3/1 compared to P3/0.1) results in a smoother and more homogeneous surface, whereas a higher poloxamer content (P4/0.1 compared to P3/0.1) leads to a rougher and uneven appearance. The addition of HC induces a more porous 3D morphology, resulting in a looser and more porous structure compared to the control samples. This alteration may influence the kinetics of drug release. Therefore, the extent of this effect likely depends on the dosage selected during the design and formulation phases.

### 2.2. Rheological Characterization

**Figure 2:**
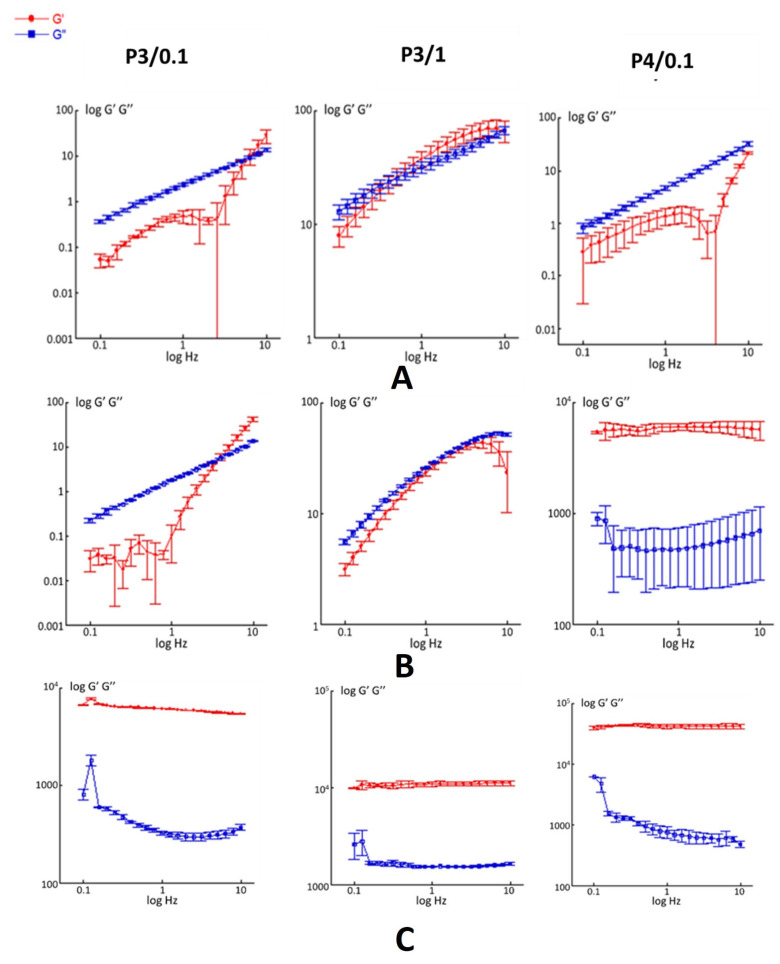
Mechanical spectra of unloaded pol/HA platforms at three different temperatures: 4 °C (**A**), 25 °C (**B**) and 37 °C (**C**).

Platforms with HA concentrations higher than 1% *w*/*v* were excluded from the study because they formed a solution whose viscosity was too high, rendering the gel unsuitable for ocular insertion, even at temperatures below 4 °C. Conversely, gels with a poloxamer concentration lower than 21.43% *w*/*v* were in a liquid state at temperatures above 37 °C and/or their gelation times were excessively long. For this reason, the study proceeded by focusing only on the P3/0.1, P3/1 and P4/0.1 formulations. Their mechanical spectra, i.e., G’ and G’’ as a function of the frequency, are shown in Figure 2.

As can be seen in Figure 2A, at 4 °C, the rheological behavior of the formulations P3/0.1 and P4/0.1 exhibited characteristics typical of a viscous fluid, i.e., G’’ is consistently higher than G’ across all the frequencies analyzed. Differently, the mechanical spectrum of the P3/1 formulation evidenced a rheological behavior typical of an entangled solution. It showed a predominantly viscous response at low frequencies and a prevalently elastic character at higher frequencies, with a crossover frequency at about 0.8 Hz. At 25 °C (Figure 2B), the rheological behavior was found to be similar for both the P3/0.1 and the P3/1 formulations, while as for the P4/0.1, which had the highest poloxamer content, both viscoelastic moduli were found to be quite constant across the entire frequency range. Notably, G’ constantly exceeded G’’, thereby showing a rheological behavior typical of a gel-like material. All three samples analyzed exhibited a gel-like behavior at 37 °C (Figure 2C), with both viscoelastic moduli approximately constant and G’ higher than G’’, over the entire frequency range analyzed. After the addition of hydrocortisone, the mechanical spectra of all the samples remained basically unchanged (Figure S2).

### 2.3. Determination of the Gelation Temperature and Time

The initial temperature and T_gel_ of the selected platforms estimated by a simple visual inspection by means of the inverted tube method are summarized in Table 2 [18].

**Figure 3:**
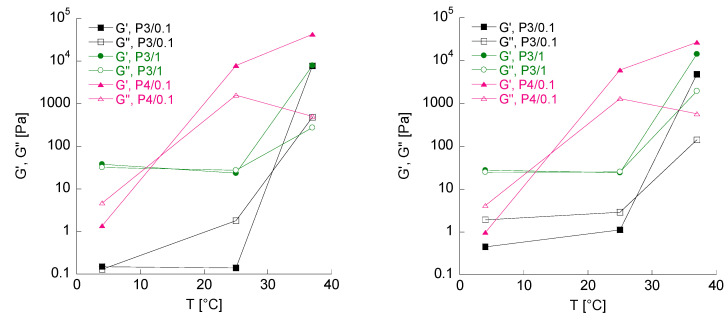
Elastic and viscous moduli as a function of temperature, at a fixed frequency of 1 Hz for formulations P3/0.1, P3/1 and P4/0.1, unloaded (**left**) and HC loaded (**right**). The error bars are omitted for clarity purpose.

A more accurate and reproducible determination of the T_gel_ of the pol/HA platforms was carried out by monitoring the variation in the elastic and viscous moduli at a fixed frequency of 1 Hz, at 4, 25 and 37 °C and under shear strain conditions where linear viscoelasticity is valid (Figure 3 and Table 3). The T_gel_ was identified as the temperature at which the sample exhibited a transition from a predominantly viscous (G’’ > G’) to a predominantly elastic behavior (G’ > G’’). In Table 3, the values of both moduli, G’ and G’’, for each formulation, at the three different analysis temperatures, are reported. As shown in Figure 3 and summarized in Table 4, the presence of HC loaded into the P3/0.1 and P4/0.1 gels did not affect their T_gel_, while the T_gel_ of the P3/1 formulation decreased to just above 30 °C. This latter platform is the most suitable for possible application, considering that the pre-corneal temperature is about 33–35 °C

### 2.4. Thermal Analyses

**Figure 4:**
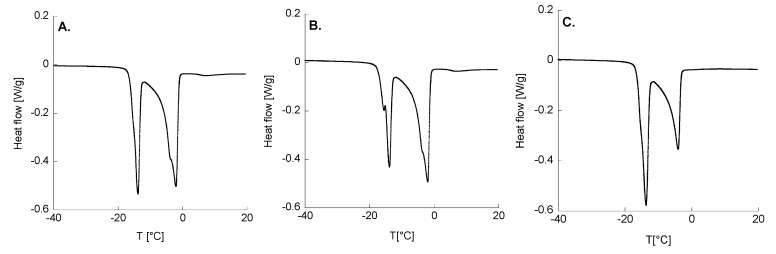
Thermograms of (**A**) P3/0.1; (**B**) P3/1; (**C**) P4/0.1.

Figure 4 displays the DSC thermograms of the formulated platforms. In all cases, two distinct endothermic peaks were detected, corresponding to the melting of the water at around −13 and −3 °C. In more detail, the first peak (around −13 °C) is associated with the fraction of water that interacts with the polymer lattice (bound water), while the second peak (around −3 °C) refers to the fraction of free water not interacting with the polymer (free water).

As can be noted from the thermograms displayed in Figure 4A,B, the DSC results evidenced that the area associated with the first peak decreased with the increasing HA content of the poloxamer gel. This finding aligns with previous studies [10,11], strongly suggesting that the addition of HA to poloxamer gels hinders the interactions between the water and poloxamer molecules.

The total heat developed during the melting of the water (ΔHtot) was calculated by integrating the two peaks, while the percentage of bound and free water was calculated as the ratio between the enthalpies associated with the first (ΔH_1_) and the second peak (ΔH_2_), normalized with respect to the actual amount of water. The total melting enthalpy (ΔH_1_ + ΔH_2_) was consistently lower than the actual melting enthalpy of the water (334 J/g) [18], indicating the presence of an “amorphous” water fraction that cannot crystallize or melt. These outcomes are summarized in Table 5 and Table 6, which show the fractions of bound and free water, as well as the melting temperature, enthalpy and onset temperature, for the P3/0.1, P3/1 and P4/0.1 formulations. The outcomes derived from the thermoanalytical analyses in this study distinctly indicate the remarkable water sequestration prowess of HA. Such a manifestation implies that the incorporation of HA within poloxamer gels effectively hampers the interactions between the water molecules and poloxamers. This discernible phenomenon implies a preferential interaction among the poloxamer moieties in the presence of HA, especially under conditions of high poloxamer concentrations.

### 2.5. Hydrogel Swelling and Release Kinetics

**Figure 5:**
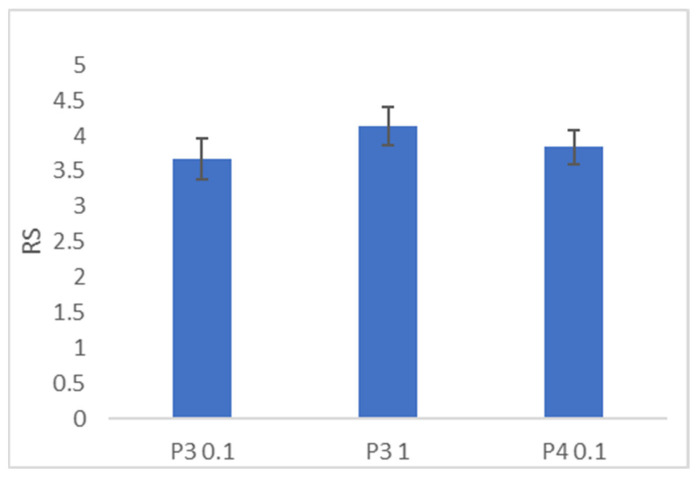
Swelling ratio of dried gel formulations.

Swelling tests were conducted to assess the water uptake capacity of the hydrogels. As illustrated in Figure 5, the swelling ratio of the hydrogel formulations ranged between 3.5 and 4. Notably, the P3/1 formulation exhibited a higher swelling ratio compared to the P3/0.1 and P4/0.1. This behavior is likely attributed to the higher percentage of HA, which is associated with the creation of a polymer matrix with larger gaps.

**Figure 6:**
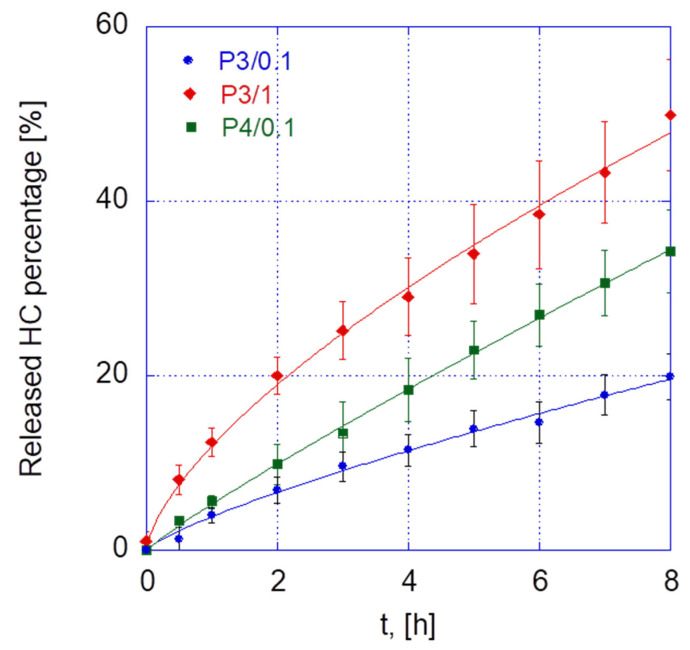
Hydrocortisone release curves from pol/HA gels.

Figure 6 shows the release profiles of the three selected platforms. In particular, the fastest release was observed in the case of the P3/1 gel, followed by the P4/0.1 and P3/0.1 formulations. In all cases, a slightly faster initial release was observed, with a forthcoming quasi-zero-order phase. It is important to note that the HC release from these gels remained incomplete within the 8 h time frame. These results support the idea that the drug release mechanisms of poloxamer gels are strongly dependent on the gel composition. Rheological studies have shown that this significantly impacts the mechanical properties of the gels, as well as their gelation temperature and time.

To further investigate the mechanisms underlying drug release, the heuristic Korsmeyer–Peppas equation was applied [19,20]:

(1) $$R_{\%}=100\;kt^{n}$$

In Equation (1), the constant k incorporates the structural and geometric characteristics of the drug release system, whereas n, also known as the diffusional or transport exponent, provides information on the potential drug release mechanisms at play. In the case of a flat geometry, as in the case of the release system used in this work, if n is 0.5, the drug release is governed solely by diffusion. The estimated parameter values are presented in Table 7. The fitting results showed the highest value of k and the lowest value of n for the P3/1 formulation. Notably, the values of n suggest that the HC release is governed by a non-purely Fickian mechanism, indicating an anomalous transport phenomenon, which is more pronounced with the addition of HA (P3/1 gel) or an increased concentration of poloxamer (P4/0.1 formulation). Thus, a higher HA content can be associated with an enhancement of purely diffusive transport compared to the underlying anomalous mechanism. It must also be underlined that during release, the gels undergo a significant dilution, which interacts with diffusion and results in higher n values for the P3/1 and P4/0.1 gels.

It must be underlined that within an 8 h time frame, which is comparable with the administration of the gel before a night’s sleep, approximately 20–50% of the drug has been released [21].

## 3. Conclusions

In this study, we have successfully developed thermosensitive polymeric platforms by blending poloxamers (F68 and F127) with low-molecular-weight hyaluronic acid (HA). These gels were carefully formulated to exhibit an appropriate gelling temperature/time and viscoelastic properties.

SEM observations revealed significant influences of both the poloxamer and HA content on the surface structure, with additional modifications induced by the hydrocortisone (HC) addition, likely impacting the drug release characteristics. Thermal analysis further supported our previous findings by confirming the role of HA in inhibiting the interaction between the poloxamers and water. The comprehensive evaluation of the rheological properties, along with gelification temperature and time, identified the P3/1 gel as the most promising candidate for an ocular drug delivery platform. However, during the in vitro drug delivery experiments, we observed an incomplete drug release, governed by an anomalous transport mechanism. To address this issue, future strategies may involve the preliminary complexation of hydrocortisone with cyclodextrins (CDs) [22]. This approach aims to discourage hydrophobic interactions between the drug and the hydrophobic segments of the poloxamers (PPOs), thereby potentially improving the drug release profiles.

Our investigation demonstrates the potential of these gels for the localized ocular delivery of hydrocortisone, leveraging their inverse rheological behavior for prolonged precorneal residence and enhanced bioavailability. By addressing the limitations of conventional dosage forms, these gels hold promise for ocular drug delivery. In summary, this work provides valuable insights into the development of thermosensitive polymeric gels for ocular drug delivery and suggests new avenues for tailored and effective ocular drug delivery platforms.

## 4. Materials and Methods

### 4.1. Materials

The poloxamers (PEOa–PPOb–PEOa), F127 (a = 100 and b = 65) and F68 (a = 76 and b = 29), were supplied by Lutrol (city, Germany). Altergon Italia s.r.l. (Morra De Sanctis (AV), Italy) kindly provided HA with a molecular weight of 830 kDa. The hydrocortisone (HC) and the salts necessary for preparing the simulated tear fluid (STF) were purchased from Sigma-Aldrich (Milano, Italy).

### 4.2. Methods

#### 4.2.1. Preparation of Polymeric Platforms

The platforms were prepared by solubilizing the two poloxamers, F127 and F68, in filtered water (with a 0.2 μm filter), using a concentration range of 15–30% *w*/*v* and 10–30% *w*/*v*, respectively. The mixture was continuously stirred at 250–300 rpm in an ice bath at 4 °C until a clear solution was obtained. Subsequently, the samples were stored overnight at 4 °C. Different concentrations of HA were added to the obtained solutions (0.1%, 1% and 2% *w*/*v*) and stirred at 300 rpm in an ice bath until the solution became clear. Then, the samples were refrigerated overnight at 4 °C. For the drug-loaded gels, hydrocortisone (HC) at a concentration of 2.5 mg/mL was incorporated into the chosen platforms by direct dispersion in the polymeric solution. Specifically, HC was added to the polymer solution at 4 °C, a temperature at which the system is liquid, indicating a pre-gel condition. The mixture was then subjected to magnetic stirring until it attained transparency.

#### 4.2.2. Measurements of pH and Osmolarity

The pH and osmolarity of the pre-gels were measured at room temperature. The pH measurements were carried out using a Seven Compact pH meter (Mettler Toledo, Toledo, OH, USA), while the osmolarity was determined with an Osmomat 030 osmometer (Gonotec, Berlin, Germany). Due to the pre-gels’ osmolarity exceeding the maximum range of the osmometer, 1:2, 1:4, 1:5, and 1:10 dilutions were performed. The osmolarity of the pre-gels was then extrapolated to obtain the osmolarity of the original samples.

#### 4.2.3. Characterization of Hydrogels

Fourier transform infrared spectroscopy (FTIR) was conducted on the raw materials and freeze-dried hydrogels (Büchi, Flawil, Switzerland; 24 h, 0.1 mbar, −60 °C) using a Jasco FT/IR 4100 spectrometer (Easton, UK). The spectra were obtained using 100 scans and a resolution of 2 cm^−1^ across the 500–4000 cm^−1^ wavenumber range at room temperature.

The morphology of the hydrogels (P3/0.1, P3/1, and P4/0.1) with and without HC was examined using a Field Emission Scanning Electron Microscope equipped with an Energy Dispersive Spectrometer (FESEM/EDS; Zeiss Merlin VP Compact, Carl-Zeiss Strasse, Oberkochen, Germany). The samples were mounted on carbon tape, metalized with an automatic sputter coater (Agar Scientific Ltd., Essex, UK), and micrographs were captured using an INCA X-stream pulse processor (Oberkochen, Germany). The settings included a 15-kV primary beam voltage, 50–100 Å filament current, variable spot size (50 to 20,000× magnification), 3 mm working distance, and 50 s real-time counting, with corrections applied using INCA Energy software 5.05.

#### 4.2.4. Rheological Characterization

The viscoelastic properties of the produced P/HA gels were evaluated in small-amplitude oscillatory shear experiments using a Kinexus rotational Malvern rheometer (Grovewood Road, Malvern, UK). A cone/plate geometry (CP4/40) was used as the measurement system. Specifically, the experiments were performed at 4, 25 and 37 °C on both unloaded and HC loaded gels, with an oscillation frequency ranging from 0.1 to 10 Hz and a strain amplitude at which linear viscosity was attained. It was thus possible to measure the shear elastic modulus (G’) and the shear loss, or viscous modulus (G’’), as a function of the frequency. G’ provides information on the elasticity or energy stored in the material during deformation, while G’’ describes the viscous character of the gels or the energy dissipated as heat. The viscoelastic moduli were measured before and after loading with HC.

#### 4.2.5. Determination of Temperature and Gelation Time

The gelation temperature of the platforms was identified by determining the variation in the elastic and viscous moduli at three different temperatures (4, 25 and 37 °C). The tests were performed at a fixed frequency of 1 Hz and at a shear strain where linear viscoelasticity was attained. Specifically, the gelation temperature (T_gel_) was identified as the temperature at which the sample showed a transition from a predominantly viscous behavior (G’’ > G’) to a predominantly elastic behavior (G’ > G’’).

#### 4.2.6. Thermodynamic Tests

Thermodynamic tests were carried out using a differential scanning calorimeter (DSC; DSC Q1000, TA Instruments, New Castle, DE, USA) in order to investigate the interactions between the water and P/HA gels. Specifically, the goal was to assess the influence of HA on the physico-chemical characteristics of the gel. For the DSC tests, the samples were accurately weighed and placed in hermetically sealed aluminum pans, which were equilibrated at −40 °C and heated to 20 °C at 1 °C/min. An inert atmosphere was maintained by a nitrogen flow at a flow rate of 50.0 mL/min. The heat involved in the melting of the water within the gels was calculated from the recorded thermograms by integrating the endothermic melting peaks with respect to the baseline of the thermograms recorded during the DSC analyses.

#### 4.2.7. Swelling Behavior

The swelling behavior of HC-loaded hydrogels was assessed gravimetrically at room temperature. The lyophilized gels were weighed and placed on plastic trays, followed by the addition of 4 mL of distilled water. The weight (mg) was monitored at predetermined time intervals, recording the maximum weight increase. The swelling ratio *S_R_* was calculated using the formula:

(2) $$S_{R}=\frac{W_{S}-W_{d}}{W_{d}}$$

where *W_S_* is the weight of the swollen hydrogels and *W_d_* is the weight of the dried hydrogels (mg).

#### 4.2.8. Hydrocortisone In Vitro Release Kinetics

The in vitro release kinetics of hydrocortisone (HC) from the optimized platforms were assessed over a period of 8 h in a simulated tear fluid (STF) solution. Sink conditions were ensured during the entire release phase [10,11]. Briefly, 1 mL of each polymeric platform containing the drug was placed in a small glass cell, equipped with a removable lid, and welded to the bottom of a larger glass cell. The cell was then filled with 40 mL of STF and immersed in a thermostatic bath at 37 °C in order to initiate the gelation of the polymeric platform. After gelation, the lid was removed to allow contact between the gel and the STF. A magnetic stirrer in the cell provided continuous stirring. At regular time intervals, 1 mL of solution was taken from the cell and replaced with an equal volume of fresh STF. The HC was quantified by spectrophotometric assay (UV-1800, Shimadzu UV spectrophotometer; λ = 247 nm). The linearity of the response was evaluated in the concentration range 0.2–16 µg/mL (R^2^ > 0.99). The method was thoroughly validated in STF at a physiological pH and in double-distilled water (DDW) at different pH values, and the results are shown in Figures S3 and S4 and summarized in Table S1.

## Figures and Tables

**Table 1:** Summary of prepared platform acronyms, with asterisks (*) denoting selected platforms. The *w*/*v* percentages refer to the added volume of water.

Acronyms	F127 (% *w*/*v*)	F68 (% *w*/*v*)	HA 830 kDa (% *w*/*v*)
P1	15	10	-
P2	15	15	-
P3	21.43	21.43	-
P4	30	30	-
P1/0.1	15	10	0.1
P1/1	15	10	1
P1/2	15	10	2
P2/0.1	15	15	0.1
P2/1	15	15	1
P2/2	15	15	2
P3/0.1 *	21.43	21.43	0.1
P3/1 *	21.43	21.43	1
P3/2	21.43	21.43	2
P4/0.1 *	30	30	0.1

**Table 2:** Gelling temperature and time of pol/HA formulations.

Formulation	T_i_ (°C)	T_gel_ (°C)	T_gel_ (min)
P3/0.1	22.0 ± 2.0	38.3 ± 0.3	8.21 ± 0.30
P3/1	22.5 ± 4.1	38.2 ± 0.4	6.33 ± 0.19
P4/0.1	20.5 ± 2.4	28.7 ± 0.5	0.15 ± 0.05

**Table 3:** Viscoelastic moduli (G’ and G’’) values of pol/HA gels at 1 Hz and different temperatures.

Formulation	T (°C)	G’ (Pa) ± SD	G’’ (Pa) ± SD
P3/0.1	4	0.15 ± 0.05	0.13 ± 0.08
	25	0.14 ± 0.04	1.80 ± 0.04
	37	(7.72 ± 1.37) × 10^3^	(4.82 ± 2.11) × 10^2^
P3/1	4	38.0 ± 6.03	32.0 ± 3.56
	25	23.5 ± 2.40	27.6 ± 0.58
	37	(7.88 ± 3.73) × 10^3^	(2.74 ± 1.32) × 10^2^
P4/0.1	4	1.38 ± 0.49	4.70 ± 0.41
	25	(7.87 ± 6.59) × 10^3^	(1.59 ± 0.57) × 10^3^
	37	(4.26 ± 0.02) × 10^4^	(5.07 ± 1.12) × 10^2^

**Table 4:** Viscoelastic moduli (G’ and G’’) values of pol/HA gels loaded with HC (2.5 mg/mL) at a fixed frequency of 1 Hz and at 4, 25 and 37 °C.

Formulation	T (°C)	G’ (Pa) ± SD	G’’ (Pa) ± SD
P3/0.1 + HC	4	0.56 ± 0.05	2.42 ± 0.08
	25	1.41 ± 2.25	3.43 ± 2.22
	37	(5.30 ± 0.52) × 10^3^	(1.71 ± 0.07) × 10^2^
P3/1 + HC	4	36.3 ± 4.78	30.8 ± 3.28
	25	26.4 ± 1.83	30.2 ± 2.15
	37	(1.49 ± 0.08) × 10^4^	(2.18 ± 0.13) × 10^3^
P4/0.1 + HC	4	1.41 ± 0.10	5.33 ± 0.11
	25	(6.63 ± 0.98) × 10^3^	(1.59 ± 0.22) × 10^3^
	37	(4.21 ± 0.12) × 10^4^	(7.20 ± 0.29) × 10^2^

**Table 5:** Percentages of free/bound and amorphous water in pol/HA gels.

Formulation	F68 (% p/V)	F127 (% p/V)	HA (% p/V)	Free H_2_O (% ± SD)	Bound H_2_O (% ± SD)	Amorphous H_2_O (% ± SD)
P3/0.1	21.43	21.43	0.1	34.8 ± 0.7	65.2 ± 0.9	32.3 ± 1.1
P3/1	21.43	21.43	1	37.9 ± 6.1	62.1 ± 6.1	40.0 ± 9.8
P4/0.1	30	30	0.1	54.2 ± 1.1	45.8 ± 1.1	36.3 ± 0.1

**Table 6:** Melting temperature, enthalpy and onset temperature values extrapolated from the two endothermic peaks of the three formulations under examination. T1 and T2 refer to the peak temperature of the first and second melting peak, respectively.

Formulation	1° Peak ENDO			2° Peak ENDO			
	T_1_ [°C]	ΔH_1_ [J/g]	T_1_ onset [°C]	T_2_ [°C]	ΔH_2_ [J/g]	T_2_ onset [°C]	ΔH tot [J/g]
P3/0.1	−13.7 ± 0.2	78.1 ± 1.1	−15.6 ± 0.1	−1.94 ± 0.11	146.6 ± 2.3	−6.32 ± 0.00	224.6 ± 3.5
P3/1	−13.8 ± 0.1	74.6 ± 0.2	−15.5 ± 0.1	−2.04 ± 0.13	124.8 ± 32.5	−6.32 ± 0.00	199.3 ± 32.7
P4/0.1	−13.8 ± 0.3	114.7 ± 2.1	−16.0 ± 0.1	−4.06 ± 0.06	96.7 ± 2.5	−6.96 ± 0.40	211.4 ± 0.4

**Table 7:** Peppas–Korsmeyer parameter estimates for HC release from P3/0.1, P3/1 and P4/0.1 gels.

	P3/0.1	P3/1	P4/0.1
*k*, h^−n^	0.0389	0.120	0.0533
*n*	0.779	0.665	0.898

## Data Availability

The data presented in this study are openly available in article.

## Supplementary Materials

The following supporting information can be downloaded at: https://www.mdpi.com/article/10.3390/gels10030193/s1, Figure S1: Measured values of osmloarity at 1:, 1:4, 1:5 and 1:10 dilutions. The values are basically the same irrespective of the HA presence; Figure S2: Mechanical spectra of pol/HA platforms loaded with HC at three different temperature 4 °C (A), 25°C (B) and 37°C (C); Figure S3: Absorbance vs. HC concentration curves at room temperature; Figure S4: Built-in becker specifically designed for release tests from thermosensitive hydrogels. (A) Interior of the becker seen from above; (B) Example of a becker containing the sample and the release medium (STF), along with the removable lid; Table S1: Slopes of the calibration curves in the linear region of Figure S3.
