# Supplementary material for: Thermosensitive In Situ Gelling Poloxamers/Hyaluronic Acid Gels for Hydrocortisone Ocular Delivery
Source: Gels. 2024 Mar 12;10(3):193. doi: 10.3390/gels10030193 (PMC10970564; doi:10.3390/gels10030193)
Supplement: Supplementary file 1 [file gels-10-00193-s001.zip › gels-2566142-supplementary.pdf]

Supplementary information

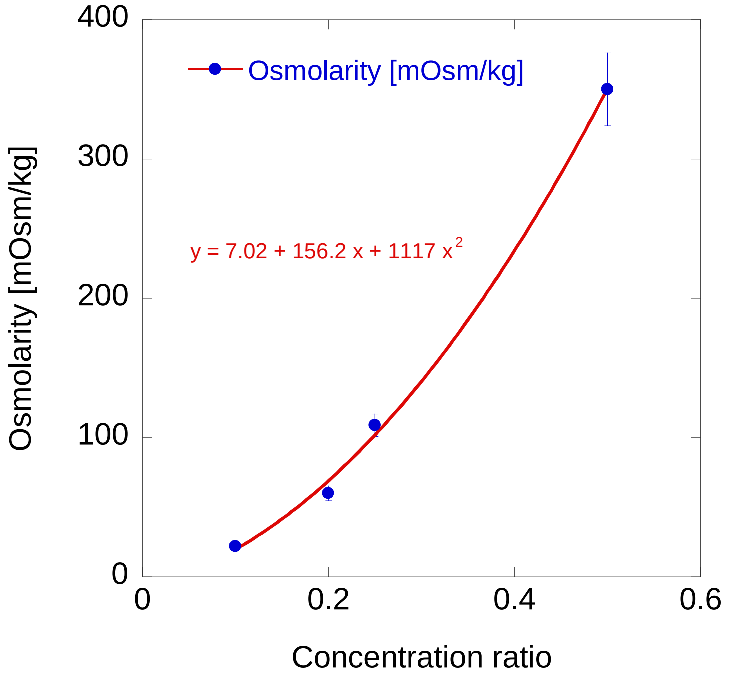

Figure S1. Measured values of osmloarity at 1:, 1:4, 1:5 and 1:10 dilutions. The values are basically the same irrespective of the HA presence.

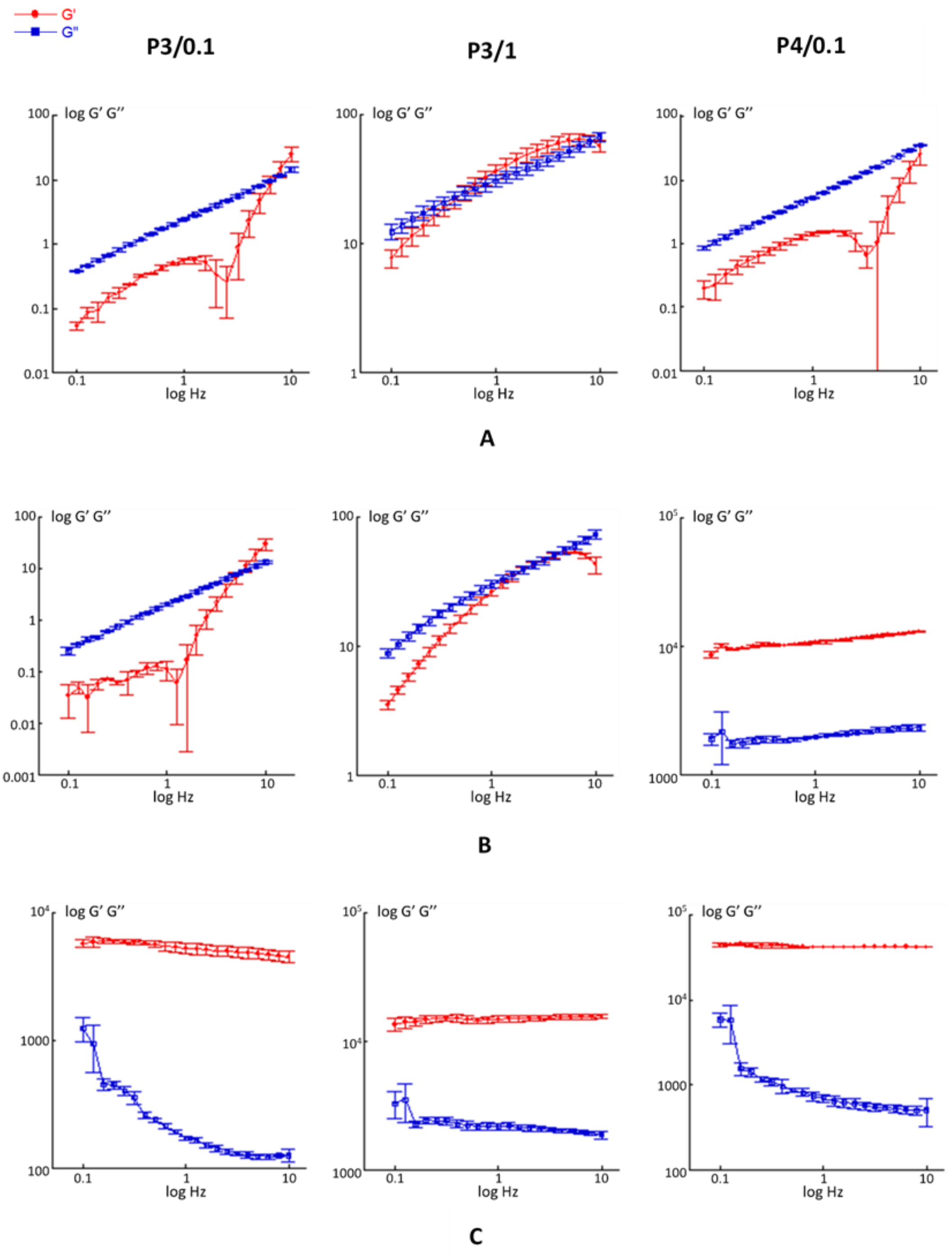

Figure S2. Mechanical spectra of pol/HA platforms loaded with HC at three different temperature 4 °C (A), 25°C (B) and 37°C (C).

.

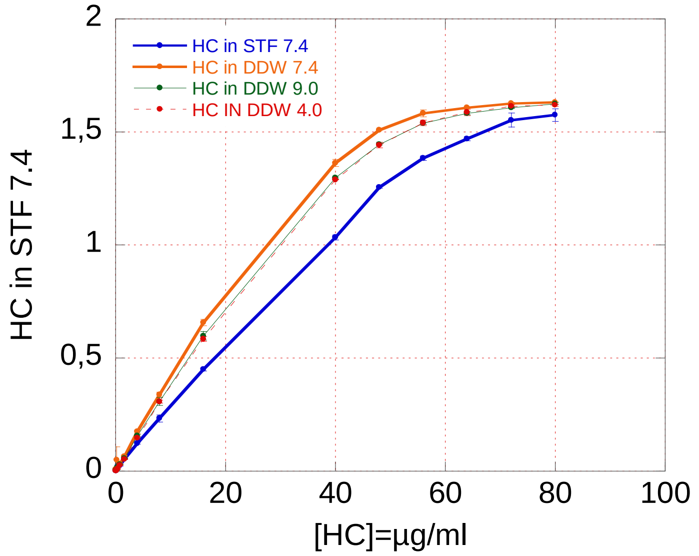

Figure S3. Absorbance vs. HC concentration curves at room temperature.

Table S1. Slopes of the calibration curves in the linear region of Figure S3.

**Calibration curves parameters**				
	**STF 7.4**	**pH 4.0**	**pH7.4**	**pH 9.0**
**Equation**	$y = 27.993 + 0.0052$	$y = 37.1165x - 0.0032$	$y = 41.287x + 0.0011$	$y = 37.559x + 0.0006$
**Slope**	27.993	37.1165	41.827	37.559
**Standard error**	0.004704	0.00677	0.00543	0.00494
**R^2**	0.9993	0.9992	0.9996	0.9996
**LOD**	0.0005 mg/ml	0.0006 mg/ml	0.00043 mg/ml	0.00043 mg/ml
**LOQ**	0.00168 mg/ml	0.00183 mg/ml	0.00131 mg/ml	0.00132 mg/ml

LOD = limit of detection

LOQ= limit of quantification

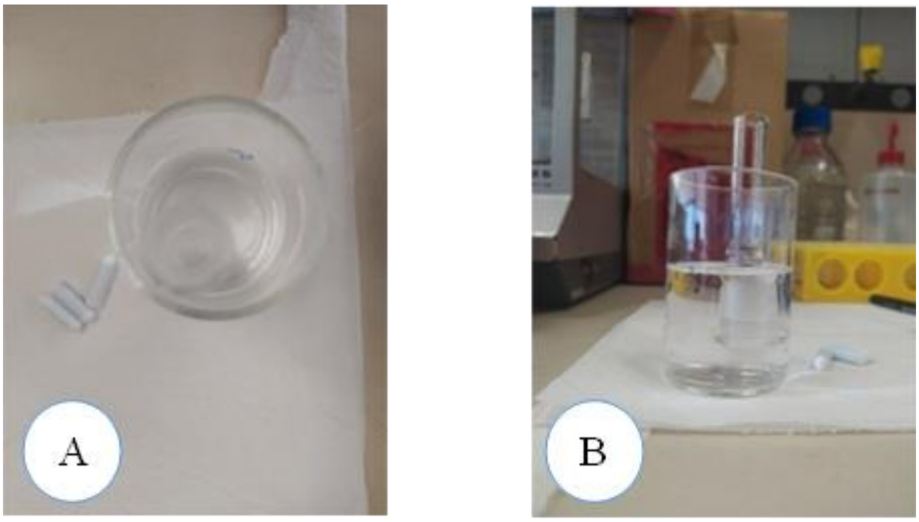

Figure S4. Built-in becker specifically designed for release tests from thermosensitive hydrogels. (A) Interior of the becker seen from above; (B) Example of a becker containing the sample and the release medium (STF), along with the removable lid.
